# Supplementary material for: An open-source framework for large-scale, flexible evaluation of biomedical text mining systems
Source: J Biomed Discov Collab. 2008 Jan 29;3:1. doi: 10.1186/1747-5333-3-1 (PMC2276192; doi:10.1186/1747-5333-3-1)
Supplement: Additional file 1 — Evaluation platform source code. The additional file contains the software evaluation framework discussed in this paper. The most current release can be downloaded from SourceForge.net [1]. See the accompanying README file for instructions on installation and use. Also included in the distribution is a collection of UIMA wrappers for some commonly used BioNLP tools and annotated corpora. [file 1747-5333-3-1-S1.zip › bionlp_uima_repository-1.0.1/docs/api/src-html/edu/uchsc/ccp/uima/annotation/CCPAnnotation_Type.html]

```
001    
002    /* First created by JCasGen Tue Aug 28 16:44:10 MDT 2007 */
003    package edu.uchsc.ccp.uima.annotation;
004    
005    import org.apache.uima.cas.FeatureStructure;
006    import org.apache.uima.cas.Type;
007    import org.apache.uima.cas.impl.CASImpl;
008    import org.apache.uima.cas.impl.FSGenerator;
009    import org.apache.uima.cas.impl.TypeImpl;
010    import org.apache.uima.jcas.JCas;
011    import org.apache.uima.jcas.JCasRegistry;
012    import org.apache.uima.jcas.tcas.Annotation_Type;
013    
014    /** The superclass for all CCP annotations.
015     * Updated by JCasGen Tue Aug 28 16:44:10 MDT 2007
016     * @generated */
017    public class CCPAnnotation_Type extends Annotation_Type {
018      /** @generated */
019      protected FSGenerator getFSGenerator() {return fsGenerator;}
020      /** @generated */
021      private final FSGenerator fsGenerator = 
022        new FSGenerator() {
023          public FeatureStructure createFS(int addr, CASImpl cas) {
024                             if (CCPAnnotation_Type.this.useExistingInstance) {
025                               // Return eq fs instance if already created
026                         FeatureStructure fs = CCPAnnotation_Type.this.jcas.getJfsFromCaddr(addr);
027                         if (null == fs) {
028                           fs = new CCPAnnotation(addr, CCPAnnotation_Type.this);
029                               CCPAnnotation_Type.this.jcas.putJfsFromCaddr(addr, fs);
030                               return fs;
031                         }
032                         return fs;
033            } else return new CCPAnnotation(addr, CCPAnnotation_Type.this);
034              }
035        };
036      /** @generated */
037      public final static int typeIndexID = CCPAnnotation.typeIndexID;
038      /** @generated 
039         @modifiable */
040      public final static boolean featOkTst = JCasRegistry.getFeatOkTst("edu.uchsc.ccp.uima.annotation.CCPAnnotation");
041    
042    
043    
044      /** initialize variables to correspond with Cas Type and Features
045            * @generated */
046      public CCPAnnotation_Type(JCas jcas, Type casType) {
047        super(jcas, casType);
048        casImpl.getFSClassRegistry().addGeneratorForType((TypeImpl)this.casType, getFSGenerator());
049    
050      }
051    }
052    
053    
054    
055
```
